# Dysphagia and Neck Swelling in a Case of Undiagnosed Lhermitte-Duclos Disease and Cowden Syndrome

**DOI:** 10.1155/2015/546297

**Published:** 2015-09-10

**Authors:** Zishuo Ian Hu, Lev Bangiyev, Roberta J. Seidman, Jules A. Cohen

**Affiliations:** ^1^Department of Medicine, Mount Sinai St. Luke's Roosevelt Hospital Center, New York, NY 10019, USA; ^2^Department of Radiology, Stony Brook University Medical Center, Stony Brook, NY 11794, USA; ^3^Department of Pathology, Stony Brook University Medical Center, Stony Brook, NY 11794, USA; ^4^Division of Hematology/Oncology, Department of Medicine, Stony Brook University Medical Center, Stony Brook, NY 11794, USA

## Abstract

We report a case of a 37-year-old woman presenting with dysphagia and thyroid masses who was subsequently diagnosed with Lhermitte-Duclos disease (LDD) based on MRI scan and histopathology. Additional imaging subsequently revealed the presence of thyroid nodules and bilateral breast cancers. Genetic testing later confirmed the diagnosis of Cowden syndrome. This case illustrates the importance of the overlap between LDD, Cowden syndrome, thyroid disease, and breast cancer.

## 1. Introduction

Somatic mutations in the phosphate and tensin homologue deleted on chromosome 10 (*PTEN*) gene have been implicated in a number of human cancers, including those of the breast, endometrium, skin, and prostate [1, 2]. PTEN predominantly functions as a tumor suppressor through its role in the phosphatidylinositol 3-kinase (PI3K) pathway [3]. By regulating PI3K signaling, PTEN inhibits the recruitment of Akt, influencing cell survival, growth, and proliferation.

Germline mutations in PTEN manifest as PTEN hamartoma tumor syndromes (PHTS), a spectrum of syndromes which includes Cowden syndrome, Bannayan-Riley-Ruvalcaba syndrome, Proteus syndrome, and Proteus-like Syndrome [4]. Cowden syndrome is an autosomal dominant syndrome characterized by multiorgan hamartomas affecting all three germ layers. Adult dysplastic gangliocytoma of the cerebellum, or Lhermitte-Duclos disease (LDD), is considered a major criterion for diagnosis of Cowden syndrome. Here, we report a case of LDD in a patient with Cowden syndrome.

## 2. Case Report

A 37-year-old East Asian woman presented with 3 days of dysphagia and 2 months of bilateral neck swelling. She denied any nausea, vomiting, headache, or gait disturbances.

The patient's family history was unknown as she was adopted. The patient's medical history was significant for bipolar disorder. On physical exam, patient was alert and oriented and cranial nerves were found to be intact. Palpable, nontender masses in the anterior neck were appreciated on head and neck examination. A mobile, nontender, 1 cm lump was also present on the left breast.

**Figure 1:**
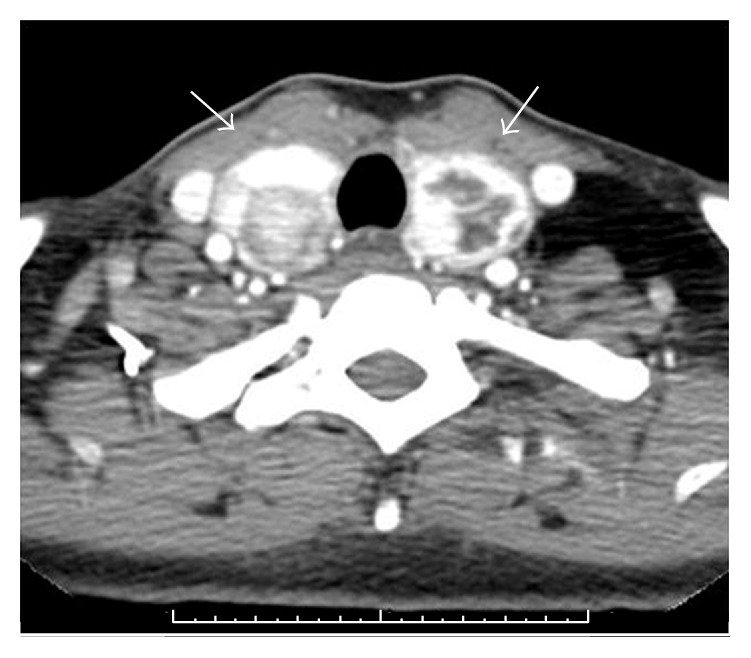
Axial postcontrast CT image through the thyroid gland demonstrates enlarged thyroid lobes that contain heterogeneously enhancing nodules bilaterally.

CT, MRI, and fine needle aspiration of the thyroid masses revealed bilateral nonmalignant nodules measuring 1.9 cm and 1.7 cm on the right side and 2.2 cm and 2.3 cm on the left (Figure 1). The nodules abutted the esophagus bilaterally.

**Figure 2:**
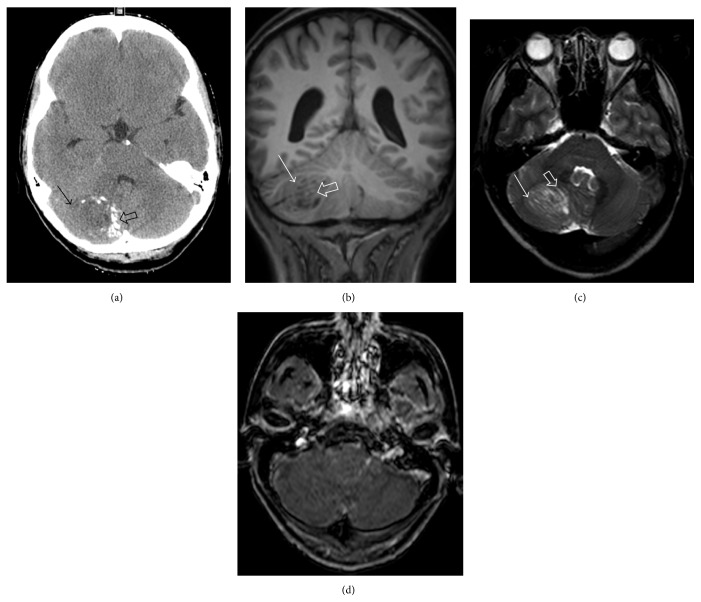
Noncontrast head CT (a) demonstrates a slightly hypodense (solid arrow) mass with calcifications (open arrow) centered in the right cerebellum. MRI of the brain demonstrates the typical striated-appearing right cerebellar mass with alternating isointense (solid arrow) and hypointense (open arrow) striations on coronal precontrast *T*_1_ weighted image (b) and isointense (solid arrow) and hyperintense (open arrow) signal on axial *T*_2_ weighted image (c). There is no appreciable enhancement of the mass on postcontrast axial *T*_1_ weighted image (d).

**Figure 3:**
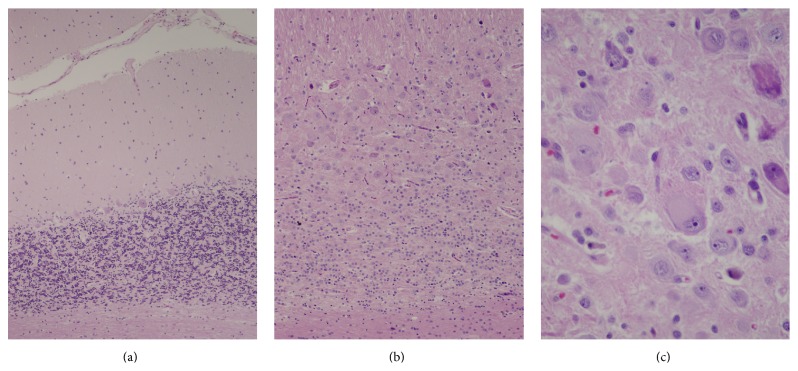
The surgical resection specimen includes a region of almost normal cerebellar parenchyma (a), with a normal granule cell layer that consists of fairly densely packed small neurons that are seen as blue nuclei separated by synaptic zones that are pink. The normal single layer of large Purkinje cells is located at the interface with the low cellularity molecular layer, here minimally more cellular than usual. Compare this with the images of the lesion (b and c). (b) The cerebellar granule cell layer in this region of the lesion is dysmorphic. Granule cells are scant and dispersed among ganglionic cells of varying sizes that expand this layer. (c) Detail of a region featured in upper right quadrant of panel (b) highlights the variable size of the abnormal ganglionic neurons characterized by relatively abundant cytoplasm and large nuclei with prominent nucleoli, some with abnormal irregularly shaped nuclei, that replace and expand the granule cell layer (hematoxylin and eosin paraffin sections; original magnifications: (a) 100x, (b) 200x, and (c) 400x).

MRI of the head revealed mild hydrocephalus and a right 3.8 cm cerebellar mass with a “tiger-stripe” appearance consistent with LDD (Figure 2). Total resection of the lesion was performed and histopathologic evaluation confirmed the diagnosis (Figure 3).

Mammogram, MRI, and core biopsy of the breasts were also performed, which showed bilateral invasive ductal carcinoma. The patient subsequently underwent bilateral mastectomy. Histology revealed a 0.8 cm invasive ductal carcinoma of the right breast (T1bN0), estrogen positive, progesterone positive, and HER2 negative, and a 1.5 cm invasive ductal carcinoma of the left breast (T1cN0), estrogen positive, progesterone positive, and HER2 negative. Her Oncotype DX breast cancer recurrence score was 24 for her left breast carcinoma, corresponding with a 15% risk of distant recurrence over 10 years. She was given 4 doses of Taxotere and Cytoxan. The patient declined tamoxifen therapy.

Genetic testing results confirmed the presence of the R335X (c.1003C>T) mutation in the PTEN gene and the patient was diagnosed with Cowden syndrome. The patient also chose to undergo risk-reducing thyroidectomy and hysterectomy with bilateral salpingectomy.

## 3. Discussion

Lhermitte and Duclos originally reported LDD in a 36-year-old man suffering from occipital headaches and diminished hearing on the left side in 1920 [5]. LDD is a hamartomatous overgrowth of the cerebellum that causes a mass effect in the posterior fossa. Patients typically present with headache, nausea, vomiting, and papilledema [6].

MRI of LDD lesions typically shows a striated appearance with hyperintense/hypointense signal on *T*_1_/*T*_2_ images [7]. Pathologically, LDD is noted for its dysplastic expansion of ganglion cells, leading to replacement of the internal granule cell layer in the cerebellum. Large neuronal cells with vesicular nuclei and prominent nucleoli are usually seen.

Lloyd and Dennis named Cowden syndrome after its first reported patient, Rachel Cowden, in 1963 [8]. About 87% of Cowden syndrome patients have a germline mutation either in the* PTEN* gene or in its promoter region [9–11]. Individuals with Cowden syndrome are at an increased risk of developing thyroid, breast, and endometrial malignancies [11, 12]. Cowden syndrome patients have a 10% lifetime risk of developing thyroid cancer [13]. Female patients have a 25% to 50% lifetime risk of developing breast cancer and an estimated lifetime risk of 5 to 10% of developing endometrial cancer [14–16]. Estimated lifetime risk for any cancer in PTEN patients has been reported as high as 89% [17].

Due to the high risk of malignancy in Cowden syndrome, the National Comprehensive Cancer Network (NCCN) recommends regular monthly breast self-exams beginning at age 18, semiannual clinical breast exams starting at age 25 or 5 to 10 years earlier than the earliest known breast cancer in the family, and annual mammograms and breast MRI screening starting at age 30–35 years or 5 to 10 years earlier than the earliest known breast cancer in the family for female patients. For women treated for breast cancer, mammography and breast MRI should be done on remaining breast tissue [18]. Annual thyroid ultrasounds should be started at time of diagnosis. Annual random endometrial biopsies or ultrasound starting at age 30–35 years are recommended for women with Cowden syndrome. In addition, colonoscopy is recommended starting at age 35 at 5-year intervals, or more often frequently when patients are symptomatic or polyps are present. Renal ultrasound can be given every one or two years after the age of 40.

A 2007 literature review found 53 reported cases of LDD associated with Cowden syndrome [19]. Derrey et al. reported a mean age of 36.7 years at the diagnosis of LDD in Cowden syndrome patients [20]. Our patient was 37 years old on presentation and found to have LDD, thyroid nodules, and bilateral breast cancers. The delay in diagnosis for our patient was likely due to underrecognition of Cowden syndrome in the clinical setting. The incidence of Cowden syndrome has been reported to be 1 in 200,000 and likely to be underestimated due to its variable expression.

Symptomatic LDD patients are recommended to have surgical resection. Thyroid cancer has the earlier onset and second greatest cumulative lifetime risks compared with other Cowden syndrome-associated malignancies [21]. For selecting patients with Cowden syndrome, including patients with developmental disorders, patients difficult to monitor routinely, and patients who have nodules and are aware of the risks and benefits of the procedure, prophylactic total thyroidectomy may be considered. Prophylactic hysterectomy should be discussed with the patient on a case-to-case basis.
